# Subtyping of early-onset Parkinson’s disease using cluster analysis: A large cohort study

**DOI:** 10.3389/fnagi.2022.1040293

**Published:** 2022-11-11

**Authors:** Zhou Zhou, Xiaoxia Zhou, Yaqin Xiang, Yuwen Zhao, Hongxu Pan, Juan Wu, Qian Xu, Yase Chen, Qiying Sun, Xinyin Wu, Jianping Zhu, Xuehong Wu, Jianhua Li, Xinxiang Yan, Jifeng Guo, Beisha Tang, Lifang Lei, Zhenhua Liu

**Affiliations:** ^1^Department of Neurology, Xiangya Hospital, Central South University, Changsha, China; ^2^Department of Geriatrics, Xiangya Hospital, Central South University, Changsha, China; ^3^National Clinical Research Center for Geriatric Disorders, Xiangya Hospital, Central South University, Changsha, China; ^4^Department of Epidemiology and Health Statistics, Xiangya School of Public Health, Central South University, Changsha, China; ^5^Hunan KeY Health Technology Co., Ltd., Changsha, China; ^6^Hunan Creator Information Technology Co., Ltd., Changsha, China; ^7^Key Laboratory of Hunan Province in Neurodegenerative Disorders, Central South University, Changsha, China; ^8^Department of Neurology, The Third Xiangya Hospital, Central South University, Changsha, China

**Keywords:** early-onset Parkinson’s disease, heterogeneous, subtype, PD-MDCNC, cluster analysis

## Abstract

**Background:**

Increasing evidence suggests that early-onset Parkinson’s disease (EOPD) is heterogeneous in its clinical presentation and progression. Defining subtypes of EOPD is needed to better understand underlying mechanisms, predict disease course, and eventually design more efficient personalized management strategies.

**Objective:**

To identify clinical subtypes of EOPD, assess the clinical characteristics of each EOPD subtype, and compare the progression between EOPD subtypes.

**Materials and methods:**

A total of 1,217 patients were enrolled from a large EOPD cohort of the Parkinson’s Disease & Movement Disorders Multicenter Database and Collaborative Network in China (PD-MDCNC) between January 2017 and September 2021. A comprehensive spectrum of motor and non-motor features were assessed at baseline. Cluster analysis was performed using data on demographics, motor symptoms and signs, and other non-motor manifestations. In 454 out of total patients were reassessed after a mean follow-up time of 1.5 years to compare progression between different subtypes.

**Results:**

Three subtypes were defined: mild motor and non-motor dysfunction/slow progression, intermediate and severe motor and non-motor dysfunction/malignant. Compared to patients with mild subtype, patients with the severe subtype were more likely to have rapid eye movement sleep behavior disorder, wearing-off, and dyskinesia, after adjusting for age and disease duration at baseline, and showed a more rapid progression in Unified Parkinson’s Disease Rating Scale (UPDRS) total score (*P* = 0.002), UPDRS part II (*P* = 0.014), and III (*P* = 0.001) scores, Hoehn and Yahr stage (*P* = 0.001), and Parkinson’s disease questionnaire-39 item version score (*P* = 0.012) at prospective follow-up.

**Conclusion:**

We identified three different clinical subtypes (mild, intermediate, and severe) using cluster analysis in a large EOPD cohort for the first time, which is important for tailoring therapy to individuals with EOPD.

## Introduction

Parkinson’s disease (PD) is the second most common neurodegenerative movement disorder characterized by typical motor symptoms and many less visible non-motor symptoms (NMSs; Poewe et al., 2017; Bloem et al., 2021). It is characterized pathologically by dopaminergic neuronal loss in the substantia nigra pars compacta and intracellular inclusions containing α-synuclein aggregates (Armstrong and Okun, 2020; Liu et al., 2022; Zhou et al., 2022). PD is a progressive and complex neurological disorder with heterogeneous symptomatology (Jafari et al., 2020; Bloem et al., 2021). Although PD is an age-related disease that typically appears after the age of 65 years, the age of onset for approximately 10% of affected individuals is younger than 50 years, referring to early-onset PD (EOPD; Zhang et al., 2005; Schrag and Schott, 2006; Poewe et al., 2017; Niemann and Jankovic, 2019). Patients with EOPD have relatively high clinical heterogeneity and a longer disease course and typically develop motor fluctuations and dyskinesias earlier, which vary dramatically in its clinical manifestations and prognosis (Pagano et al., 2016; Schirinzi et al., 2020). Thus, EOPD requires more personalized treatment and long-term management.

Several previous studies have used cluster analysis to define clinical PD subtypes based on motor severity, motor complications, some non-motor features, and demographic characteristics (Fereshtehnejad et al., 2015; Lawton et al., 2018; De Pablo-Fernández et al., 2019; Belvisi et al., 2021; Brendel et al., 2021; Mestre et al., 2021). Growing evidences have shown that there are distinct subtypes of PD with diverging trends of progression (Qian and Huang, 2019; Hendricks and Khasawneh, 2021; Mestre et al., 2021). However, all previous cluster analyses were limited to patients with PD, and an EOPD cluster analysis was not available. With some patients with EOPD following a relatively benign course and others progressing rapidly to disability, subtyping EOPD is required. It is essential to perform cluster analysis based on deep phenotyping, followed by prospective validation of subtypes. Defining different subcategories of EOPD is key to better understand its underlying disease mechanisms, predict its disease course, and subsequently design more efficient personalized management strategies (Qian and Huang, 2019).

This study aimed to (1) identify clinical EOPD subtypes using cluster analysis based on a comprehensive baseline dataset, (2) assess the clinical characteristics of each EOPD subtype, and (3) compare disease progression between different EOPD subtypes.

## Materials and methods

### Participants

Participants were enrolled from a large EOPD cohort of the Parkinson’s Disease & Movement Disorders Multicenter Database and Collaborative Network in China (PD-MDCNC) between January 2017 and September 2021. The clinical diagnosis of PD was confirmed by at least two neurological specialists according to the Movement Disorder Society Clinical Diagnostic Criteria for Parkinson’s Disease (Postuma et al., 2015), including diagnoses of either clinically established or probable PD. The exclusion criteria were as follows: (1) familial history, (2) missing data ≥ 10%, and (3) diagnosis of other causes of parkinsonism on baseline or follow-up assessments. The clinical data of all participants were stored in the PD-MDCNC.^1^ Written informed consent was obtained from all the participants. This study was approved by the Ethics Committee of Xiangya Hospital and was conducted in accordance with the ethical guidelines of the Declaration of Helsinki.

### Clinical assessment

All participants underwent comprehensive and standardized clinical assessments. Before conducting the assessment, all researchers were trained to ensure an equal understanding of the scales used and the methods and phrasing for clinical data collection. Demographic information and clinical characteristics were collected, as described in our previous study (Zhao et al., 2020; Zhou et al., 2022). Clinical examinations of motor symptoms were performed based on the Unified Parkinson’s Disease Rating Scale (UPDRS) and Hoehn and Yahr (H&Y) stages, defining motor subtypes as either tremor dominant, postural instability/gait difficulty or indeterminate group. Motor complications, such as dyskinesia and wearing-off were diagnosed by clinicians, and the severities of dyskinesia and wearing-off were evaluated by UPDRS part IV-A and 9-item End-of-dose Wearing-off Questionnaire (WOQ-9), respectively. Freezing of gait (FOG) was evaluated using New Freezing of Gait Questionnaire (NFOGQ).

In addition to motor symptoms, we evaluated a broad range of NMSs based on the NMSS, the Scale for Outcomes in Parkinson’s Disease for Autonomic Dysfunction (SCOPA-AUT), Mini-Mental State Examination (MMSE), Rapid Eye Movement Sleep Behavior Disorder Questionnaire-Hong Kong (RBDQ-HK), Epworth Sleepiness Scale (ESS), Parkinson’s Disease Sleep Scale (PDSS), Hyposmia Rating Scale (HRS), Functional Constipation Diagnostic Criteria Rome III, and Hamilton Depression Scale (HAMD-17). Quality of life was assessed using the Parkinson’s disease questionnaire-39 item version (PDQ-39). Details regarding the clinical scales were provided in our previous study (Zhou et al., 2022). Patients with illiteracy, primary education, and above junior education were identified as having cognitive impairment when the MMSE scores were below 17, 20, and 24 points, respectively. Hyposmia was defined as a total HRS score less than 22.5. Rapid eye movement sleep behavior disorder (RBD) was defined as a total RBDQ-HK scale score no less than 18. Excessive daytime sleepiness (EDS) was defined as a total ESS score higher than 10. Depression was defined as a total HAMD-17 score higher than 7. The levodopa equivalent daily dose (LEDD) was calculated based on a commonly used method (Tomlinson et al., 2010).

After a mean follow-up period of 1.5 years, the same patients were reassessed on the same variables as the baseline.

### Database

Our team established the PD-MDCNC, a comprehensive yet flexible, user friendly, secure, and easily accessible database. And we launched the Chinese Early-Onset Parkinson’s Disease Registry (CEOPDR), which is a large and longitudinal study designed to assess clinical features, genetic architecture, imaging, and biologic markers of EOPD progression in China. All study data will be integrated in the CEOPDR study database through the PD-MDCNC.

### Data preprocessing

We conducted standardization to eliminate the influence of various dimensions by scaling the variables to zero mean and unit variance. Correlation analysis was performed between the two variables. When the calculated coefficient was greater than 0.98, only one feature was retained. Finally, the analysis did not exclude any factors. To address high-dimensional and multicollinearity problems, we used principal component analysis (PCA). PCA was performed using the Python software (version 3.6).

### Cluster analysis

All data downloaded from the PD-MDCNC database were analyzed using R version 4.1.2.^2^ Agglomerative hierarchical clustering, K-means clustering, and spectral clustering analyses were synchronously performed. We computed the Calinski–Harabasz score to estimate the optimal clustering methods. Ultimately, agglomerative hierarchical clustering was performed because of the higher Calinski–Harabasz score and better-balanced data distribution (Supplementary Tables 1, 2). Visualization of the final hierarchical cluster solution was performed using Python software (version 3.6) (Supplementary Figure 1). A flowchart of data-driven clustering is shown in Supplementary Figure 2.

### Statistical analyses

For the analysis of cross-sectional data, continuous variables were analyzed using one-way analysis of variance or non-parametric tests. Categorical variables were analyzed using the chi-squared test. Comparison of the baseline demography and clinical features between the three statistical clusters was also applied, adjusting for age and disease duration (continuous variables were analyzed by linear regression, and categorical variables were analyzed by logistic regression model).

We used general linear models (GLMs) for a comprehensive longitudinal comparison of the progression of the three subtypes. In each GLM, change of clinical characteristics was defined as the dependent variable. To reduce the regression toward the mean bias, the analysis was adjusted by the follow-up duration and baseline values of the clinical factors (Vickers and Altman, 2001; Fereshtehnejad et al., 2017). Statistical significance was defined as *P* < 0.05. All data were analyzed using the IBM SPSS Statistics version 23.0 (IBM Corp., Armonk, NY, USA).

## Results

### Overview

A total of 1,217 patients with EOPD were included in this study. The mean age was 50.54 ± 6.82 years, 53.66% were male patients, and the mean age at onset was 44.12 ± 5.54 years, with an average disease duration of 6.34 ± 5.22 years. The mean UPDRS part I, II, III, and total scores were 2.28 ± 1.98, 10.98 ± 6.56, 25.42 ± 15.73, and 40.58 ± 23.25, respectively. Among the entire study population, RBD and dementia were found in 322 (26.46%) and 103 (8.46%) patients, respectively, at baseline. Supplementary Table 3 summarizes the baseline clinical characteristics of the patients.

### Cluster results and baseline characteristics in different clusters

**FIGURE 1:**
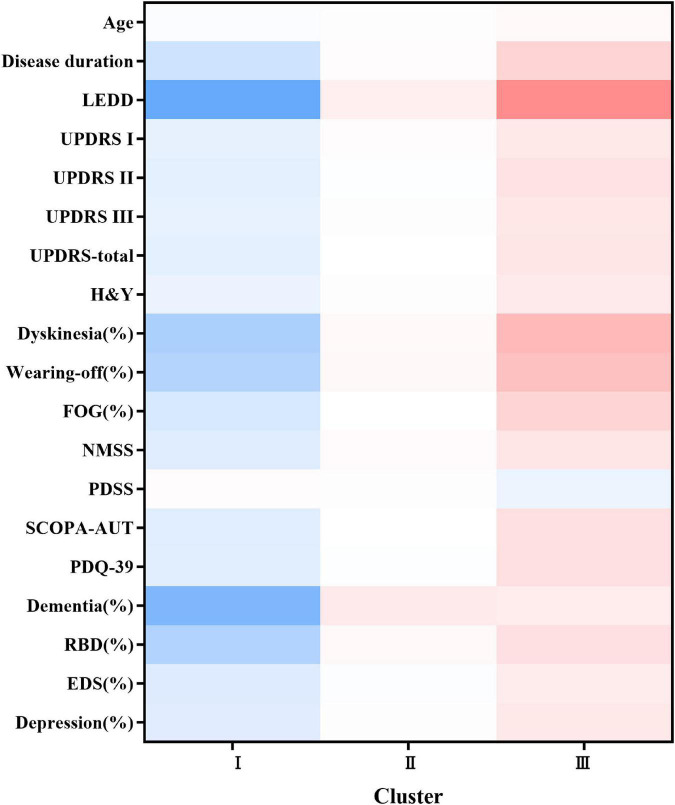
Heatmap of the three subtypes at baseline. The figure was depicted according to the mean values or percentages of subtypes. The red color represented a more severe deficit and the blue color referred to a less severe symptom. The darker the figure described, the larger difference were shown among the subtypes. Variables with *P* value <0.05 are shown. LEDD, Levodopa Equivalent Daily Dose; UPDRS, Unified Parkinson’s disease Rating Scale; H&Y, Hoehn and Yahr; FOG, freezing of gait; NMSS, Non-Motor Symptoms Scale; PDSS, Parkinson’s Disease Sleep Scale; SCOPA-AUT, the Scale for Outcomes in Parkinson’s Disease for Autonomic Dysfunction; PDQ-39, Parkinson’s disease questionnaire-39 item version; RBD, Rapid Eye Movement Sleep Behavior Disorder; EDS, excessive daytime sleepiness.

The following 25 variables were included in the final clustering solution: age, age at onset, sex, duration, body mass index, LEDD, UPDRS total score, UPDRS part I–III scores, NMSS score, PDSS score, SCOPA-AUT score, PDQ-39 score, H&Y stage score, motor subtypes, dyskinesia, wearing-off, FOG, RBD, depression, EDS, dementia, hyposmia, and constipation (Supplementary Table 3). As illustrated in Supplementary Figure 1 (visualization of the final hierarchical cluster solution in the EOPD cohort), cluster analysis revealed three distinct clusters of patients with EOPD. Detailed characteristics of the three clusters are listed in Table 1. We also demonstrated the discriminative power of these features using the heatmap shown in Figure 1. We observed evident baseline differences in motor and non-motor manifestations among clusters, with clinically important effect sizes.

**TABLE 1:** Comparison of the baseline demography and clinical features between the three clusters of EOPD cohort based on hierarchical clustering solution.

Characteristic	Cluster*			*P*-value	Adjusted *P*-value**	Multiple comparisons***
				
	I (*n* = 533)	II (*n* = 512)	III (*n* = 172)			
Age	49.54 ± 6.59	50.67 ± 6.86	53.22 ± 6.70	<0.001	−	–
Gender ratio (male,%)	52.16	53.52	58.72	0.323	0.158	–
BMI	22.66 ± 3.01	22.54 ± 3.16	22.51 ± 2.77	0.715	0.880	–
Age at onset	44.24 ± 5.77	44.13 ± 5.32	43.76 ± 5.49	0.325	0.413	-
Disease duration	5.20 ± 4.83	6.48 ± 5.14	9.44 ± 5.30	<0.001	−	–
LEDD	124.40 ± 85.88	367.62 ± 74.17	766.12 ± 197.62	<**0.001**	<**0.001**	**All comparisons**
UPDRS part I	2.08 ± 1.88	2.31 ± 2.02	2.82 ± 2.03	<0.001	0.140	–
UPDRS part II	9.92 ± 5.78	10.89 ± 6.63	14.52 ± 7.41	<**0.001**	<**0.001**	I **vs.** III, II **vs.** III
UPDRS part III	23.31 ± 14.16	25.44 ± 15.78	31.89 ± 18.29	<**0.001**	**0.034**	I **vs.** III
UPDRS total score	36.76 ± 20.71	40.54 ± 23.48	52.49 ± 25.96	<**0.001**	**0.001**	I **vs.** III, II **vs.** III
TD/Indeterminate/PIGD (%)	27.02/19.89/53.09	27.54/16.80/55.66	20.35/13.95/65.70	0.051	−	–
H&Y	1.97 ± 0.83	2.15 ± 0.87	2.58 ± 0.88	<**0.001**	**0.001**	I **vs.** III, II **vs.** III
Dyskinesia (%)*^a^*	12.95	20.12	34.88	<**0.001**	**0.001**	**All comparisons**
Wearing-off (%)*^b^*	19.51	28.91	47.09	<**0.001**	<**0.001**	**All comparisons**
FOG (%)*^c^*	23.08	26.95	40.12	<0.001	0.352	–
NMSS	27.71 ± 26.12	31.94 ± 28.59	39.26 ± 27.19	<0.001	0.165	–
PDSS	121.75 ± 26.82	119.76 ± 25.78	111.04 ± 26.35	<0.001	0.099	–
SCOPA-AUT	5.75 ± 5.35	6.42 ± 6.02	8.72 ± 6.95	<**0.001**	**0.013**	I **vs.** III, II **vs.** III
PDQ-39	24.95 ± 22.65	27.53 ± 25.70	37.73 ± 26.53	<0.001	0.051	–
Dementia (%)*^d^*	5.63	10.74	10.47	0.007	0.052	–
RBD (%)*^e^*	20.45	27.93	40.70	<**0.001**	**0.029**	I **vs.** III
EDS (%)*^f^*	22.89	25.37	32.56	0.038	0.503	–
Hyposmia (%)*^g^*	31.71	34.18	38.95	0,210	0.947	–
Depression (%)*^h^*	24.58	27.54	36.05	0.014	0.156	–
Constipation (%)*^i^*	16.28	18.95	24.55	0.055	0.978	–

*Quantitative data were expressed as mean ± SD, categorical variables were expressed as percentages, unless otherwise indicated. **Adjusted *P*-value were performed between subtypes comparisons by multivariable linear or logistic regression (controlled for baseline age and duration of disease). ***Multiple comparisons were performed among the three subtypes if adjusted *P*-value was less than 0.05. Significant *P*-values are indicated in bold. ^*a*–*i*^Evaluated, respectively by UPDRS part IV-A, WOQ-9, the 9-item End-of-dose Wearing-off Questionnaire; NFOGQ, New Freezing of Gait Questionnaire; MMSE, Mini-Mental State Examination; RBDQ-HK, Rapid Eye Movement Sleep Behavior Disorder Questionnaire-Hong Kong; ESS, Epworth Sleepiness Scale; HRS, Hyposmia Rating Scale; HAMD-17, Hamilton Depression Scale and Rome III criteria; EOPD, Early-onset Parkinson’s Disease; BMI, Body Mass Index; LEDD, Levodopa Equivalent Daily Dose; UPDRS, Unified Parkinson’s disease Rating Scale; TD, Tremor-Dominant; PIGD, Postural Instability and Gait Difficulty; H&Y, Hoehn and Yahr; FOG, freezing of gait; NMSS, Non-Motor Symptoms Scale; PDSS, Parkinson’s Disease Sleep Scale; SCOPA-AUT, the Scale for Outcomes in Parkinson Disease for Autonomic Dysfunction; PDQ-39, Parkinson’s disease questionnaire-39 item version; RBD, Rapid Eye Movement Sleep Behavior Disorder; EDS, excessive daytime sleepiness.

The first cluster of 533 patients (cluster I, termed mild motor and non-motor dysfunction based on baseline features) was characterized by a low frequency of RBD, wearing-off, and dyskinesia and mild motor and NMSs. Motor symptoms/signs were relatively mild, with the lowest mean UPDRS part II and, III and total scores (*P* < 0.05). The H&Y stage of the patients was relatively mild. RBD [109 (20.45%) patients], wearing-off [104 (19.51%) patients], and dyskinesia [69 (12.95%) patients] were uncommon. Autonomic symptoms were generally mild (5.75 ± 5.35 in the SCOPA-AUT scale, *P* = 0.013). Moreover, the average LEDD was lower in cluster I than in clusters II and III (*P* < 0.001).

At the other extreme, the third cluster of 172 patients (cluster III, termed severe motor and non-motor dysfunction based on baseline features) was characterized by a high frequency of RBD, wearing-off and dyskinesia and more severe motor and NMSs. Motor symptoms/signs were relatively severe, with the highest mean UPDRS part II and, III and total scores (*P* < 0.05). The H&Y stage score were also relatively worse. RBD [70 (40.70%) patients], wearing-off [81 (47.09%) patients], and dyskinesia [60 (34.88%) patients] were more common in cluster III than in clusters I and II (*P* < 0.05). Autonomic symptoms were the most severe (8.72 ± 6.95 in the SCOPA-AUT scale, *P* = 0.013). Moreover, the average LEDD was significantly higher in cluster III than in clusters I and II (*P* < 0.001).

The patients in cluster II (512 patients with the subtype of intermediate EOPD) had intermediate motor and NMSs between clusters I and III. The UPDRS part II, III, and total scores were intermediate. The H&Y stage of the patients was relatively moderate. RBD (143 [27.93%] patients), wearing-off (148 [28.91%] patients) and dyskinesia (103 [20.12%] patients) were moderately frequent.

### Disease progression in different clusters

**FIGURE 2:**
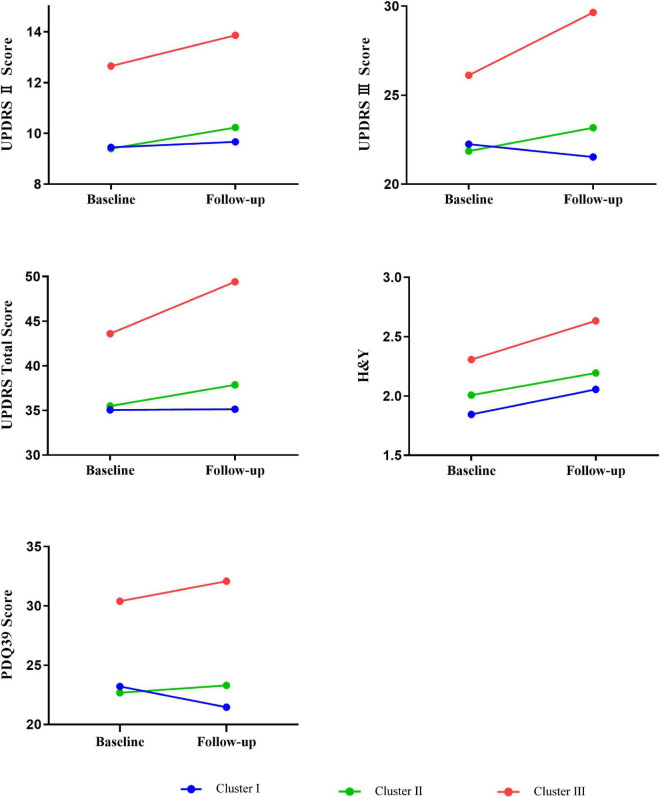
Longitudinal changes in outcomes of interest in different phenotypes of EOPD at follow-up. In each general linear models, change of clinical characteristics was defined as the dependent variable, and was adjusted by the follow-up duration and baseline value of clinical factors. Mean follow-up duration in the entire population = 1.5 years. UPDRS, Unified Parkinson’s disease Rating Scale; H&Y, Hoehn and Yahr; PDQ-39, Parkinson’s disease questionnaire-39 item version.

After a mean duration of 1.5 years, follow-up data were available for 454 patients (Table 2 and Figure 2). Patients in cluster III had a dramatically worse prognosis, with a more rapid progression in the UPDRS total (*P* = 0.009), UPDRS part II (*P* = 0.035), UPDRS part III (*P* = 0.004), H&Y stage (*P* = 0.006), and PDQ-39 (*P* = 0.037) scores. The intermediate cluster had a medium progression rate, which was slightly higher than that of cluster I.

**TABLE 2:** Longitudinal changes in clinical motor and non-motor outcomes in three different clinical phenotypes of EOPD at follow-up.

Outcome	Phenotype			Total *P*-value	*P*-value
			
	I (*n* = 216)	II (*n* = 181)	III (*n* = 57)		
UPDRS total score					
t2-t1	0.50 (17.04)	2.38 (16.11)	5.82 (18.65)	**0.009**	–
β adjusted coefficient (95% CI)	0*	2.24 (−0.95 to 5.43)	7.44 (2.67 to 12.20)	–	***P***_II_ **= 0.168**, ***P***_III_ **= 0.002**
UPDRS part I score					
t2-t1	0.06 (2.06)	0.05 (2.08)	0.49 (2.46)	0.079	–
β adjusted coefficient (95% CI)	0*	0.13 (−0.24 to 0.50)	0.64 (0.08 to 1.19)	–	*P*_II_ = 0.489, *P*_III_ = 0.024
UPDRS part II score					
t2-t1	0.21 (4.30)	0.83 (4.76)	1.21 (5.18)	**0.035**	–
β adjusted coefficient (95% CI)	0*	0.67 (−0.21 to 1.55)	1.67 (0.34 to 2.99)	–	***P*_II_ = 0.136**, ***P*_III_ = 0.014**
UPDRS part III score					
t2-t1	−0.72 (12.45)	1.31 (12.36)	3.53 (13.77)	**0.004**	–
β adjusted coefficient (95% CI)	0*	2.08 (−0.23 to 4.39)	5.63 (2.19 to 9.06)	–	***P*_II_ = 0.078**, ***P*_III_ = 0.001**
H&Y					
t2-t1	0.21 (0.73)	0.19 (0.74)	0.30 (0.67)	**0.006**	–
β adjusted coefficient (95% CI)	0*	0.05 (−0.071 to 0.18)	0.31 (0.123 to 0.50)	–	***P*_II_ = 0.391**, ***P*_III_ = 0.001**
NMSS					
t2-t1	0.61 (24.03)	0.91 (23.97)	3.49 (31.08)	0.335	–
β adjusted coefficient (95% CI)	0*	1.92 (−2.76 to 6.60)	5.00 (−1.86 to 11.88)	–	*P*_II_ = 0.420, *P*_III_ = 0.153
PDSS					
t2-t1	−4.27 (27.89)	−3.33 (28.65)	−4.69 (27.66)	0.866	–
β adjusted coefficient (95% CI)	0*	0.48 (−4.98 to 5.95)	−1.72 (−9.66 to 6.21)	–	*P*_II_ = 0.862, *P*_III_ = 0.670
SCOPA-AUT					
t2-t1	1.38 (5.82)	1.54 (6.15)	1.85 (5.11)	0.707	–
β adjusted coefficient (95% CI)	0*	0.51 (−0.79 to 1.81)	0.56 (−1.49 to 2.61)	–	*P*_II_ = 0.443, *P*_III_ = 0.589
PDQ-39					
t2-t1	−1.10 (19.92)	1.47 (22.10)	4.07 (23.59)	**0.037**	–
β adjusted coefficient (95% CI)	0*	2.48 (−1.49 to 6.46)	7.63 (1.70 to 13.55)	–	***P*_II_ = 0.220**, ***P*_III_ = 0.012**
MMSE					
t2-t1	0.17 (2.17)	0.32 (2.40)	−0.30 (2.17)	0.249	–
β adjusted coefficient (95% CI)	0*	0.00 (−0.42 to 0.43)	−0.52 (−1.15 to 0.12)	–	*P*_II_ = 0.988, *P*_III_ = 0.114
RBDQ-HK					
t2-t1	1.53 (11.51)	1.45 (12.78)	2.56 (16.21)	0.355	–
β adjusted coefficient (95% CI)	0*	0.25 (−2.27 to 2.772)	2.69 (−1.04 to 6.43)	–	*P*_II_ = 0.846, *P*_III_ = 0.157
ESS					
t2-t1	−0.07 (6.03)	0.08 (6.55)	1.22 (5.84)	0.156	–
β adjusted coefficient (95% CI)	0*	0.82 (−0.31 to 1.96)	1.44 (−0.25 to 3.13)	–	*P*_II_ = 0.156, *P*_III_ = 0.095
HRS					
t2-t1	−1.28 (4.62)	−1.12 (4.77)	−1.40 (5.66)	0.998	–
β adjusted coefficient (95% CI)	0*	−0.02 (−0.98 to 0.94)	−0.05 (−1.44 to 1.35)	–	*P*_II_ = 0.968, *P*_III_ = 0.947
HAMD					
t2-t1	0.54 (5.85)	0.34 (4.80)	1.06 (6.81)	0.945	–
β adjusted coefficient (95% CI)	0*	0.17 (−0.88 to 1.21)	0.17 (−1.39 to 1.72)	–	*P*_II_ = 0.752, *P*_III_ = 0.834

All presented values are mean (standard deviation), unless otherwise specified. In each general linear models, change of clinical characteristics was defined as the dependent variable, and was adjusted by the follow-up duration and baseline value of clinical factors. *Reference group. Significant *P*-values are indicated in bold. EOPD, Early-onset Parkinson’s Disease; UPDRS, Unified Parkinson’s Disease Rating Scale; H&Y, Hoehn and Yahr; NMSS, Non-Motor Symptoms Scale; PDSS, Parkinson’s Disease Sleep Scale; SCOPA-AUT, the scale for outcomes in Parkinson’s Disease for Autonomic Dysfunction; PDQ-39, Parkinson’s disease questionnaire-39 item version; MMSE, Mini Mental State Examination; RBDQ-HK, Rapid Eye Movement Sleep Behavior Disorder Questionnaire-Hong Kong; ESS, Epworth Sleepiness Scale; HRS, Hyposmia Rating Scale; HAMD, Hamilton Depression Scale.

Results from the GLM adjusted for baseline values and follow-up duration showed that compared to the cluster I, the cluster III subtypes had significantly greater progression in UPDRS total score (7.44 units more increase in compared to the mild subtype), UPDRS part II score (1.67 points more decline), UPDRS part III score (5.63 points more decline), H&Y stage score (0.31 points more decline), and PDQ-39 score (7.63 points more decline), which demonstrated the worst prognosis of all groups. Similar hierarchical progression was also observed in several non-motor features, namely, NMSS (1.92 points faster in cluster II, 5.00 points faster in cluster III), SCOPA-AUT (0.51 and 0.56 points faster), RBDQ-HK (0.25 and 2.69 points faster), ESS (0.82 and 1.44 points faster), HRS (−0.02 and −0.05 points faster), and HAMD (0.17 and 0.17 points faster) scores. Nevertheless, all showed no statistical significance.

However, the rate of progression was not statistically different between clusters I and II. As illustrated in Figure 2, the faster slope of progression in cluster III was most observed for the UPDRS total, UPDRS part III, and PDQ-39 scores. Based on this prognostic information, we updated the terminology of cluster III to severe motor and non-motor dysfunction/malignant and that of cluster I to mild motor and non-motor dysfunction/slow progression, leaving cluster II terminology unchanged (i.e., intermediate). The data distribution of all clusters and the prevalence of clinical symptoms at baseline and follow-up are shown in Supplementary Figures 3, 4, respectively.

## Discussion

To the best of our knowledge, this is the first study to explore the classification of patients with EOPD with a longitudinal large-sample cohort in a comprehensive database on a broad spectrum of motor and non-motor characteristics. Our study found that the most critical determinants of EOPD subtype and prognosis were motor and some NMSs, especially the UPDRS total score, motor complications, RBD, and autonomic dysfunction. Three subtypes were identified: mild motor and non-motor dysfunction/slow progression (cluster I), intermediate (cluster II) and severe motor and non-motor dysfunction/malignant (cluster III). Identification of these subtypes at baseline helps predict prognosis.

Evidence suggests that early-onset Parkinson’s disease is clinically heterogeneous; however, cluster studies of clinical subtypes in patients with EOPD remain scarce. We compared different clustering solutions in our EOPD cohort and selected the best solution to compare the baseline differences and disease progression among the clusters. As expected, baseline differences in motor and NMSs were observed among the clusters. The mild subtype represents patients with EOPD who have mild motor and non-motor manifestations, and RBD and motor complications might be present but are milder than those present in cluster III. These patients had the most favorable disease course, with the least worsening of the UPDRS total score after 1.5 years. In contrast, the severe subtype had a high frequency of RBD, wearing-off, and dyskinesia at baseline. These patients also exhibited more severe motor and autonomic symptoms. This subtype showed the most rapid and malignant progression rate in terms of the UPDRS total, UPDRS part II, UPDRS part III, H&Y stage, and PDQ-39 total scores. Between these two extremes, the intermediate subtype was defined as having intermediate motor and NMSs. This subtype showed a moderate progression rate, which was slightly higher than that in cluster I. Our results further confirmed the clinical heterogeneity of EOPD. Defining EOPD subtypes contributes to a better understanding of the underlying mechanisms of EOPD, predicts the disease course of EOPD, and leads to tailored treatment strategies (Hendricks and Khasawneh, 2021).

A heterogeneous clinical presentation and prognosis of patients with PD is increasingly recognized; in a recent longitudinal study, the presence of RBD, cognitive impairment, and autonomic dysfunction were the best predictors of a diffuse/malignant phenotype of PD (Fereshtehnejad et al., 2015). In our study, patients with the severe subtype also showed a higher propensity for RBD and more worsened autonomic disturbance, in agreement with previous studies (Fereshtehnejad et al., 2015, 2017). Previous study (Fereshtehnejad et al., 2015, 2017; Udow et al., 2016; Jozwiak et al., 2017) also found that these NMSs often coincided, suggesting a common neurobiological factor (Udow et al., 2016). Reduced noradrenergic function in PD was associated with the presence of RBD and autonomic dysfunction and noradrenergic impairment may contribute to the high prevalence of these NMSs in PD (Sommerauer et al., 2018). In addition, compared to patients with mild and intermediate EOPD subtypes, patients with severe EOPD subtype had more severe motor symptoms and higher frequency of motor complications in our study, which are consistent with the results of other similar studies in PD (van Rooden et al., 2011; Belvisi et al., 2021), and severe motor and non-motor dysfunction/malignant subtype may indicate a relatively diffuse neurodegenerative process. Different from the results of other cohort studies, our study further highlights the importance of motor complications as drivers of EOPD subtyping and prognosis.

The mechanism underlying the EOPD subtype differences remains unclear. Studies have indicated that genetic, environmental factors, gene-environment interactions, or all of these factors may play an important role in the pathogenesis of EOPD. There is accumulating evidence indicating that different genotypes of EOPD may lead to distinct clinical characteristics and rate of progression. For example, EOPD with *PRKN*, *PINK1*, or *DJ-1* mutations are characterized by good response to L-dopa treatment, dystonia and dyskinesia being relatively common, cognitive decline relatively uncommon and a slower deterioration of the disease when compared with idiopathic PD (Kasten et al., 2018; Lin et al., 2019; Zhao et al., 2020; Sun et al., 2021). Patients of EOPD with *SNCA* mutations show asymmetric onset, good responsiveness for L-dopa in initial time, early motor complications, rapid progression and worse cognitive impairment (Trinh et al., 2018; Chen et al., 2020; Zhao et al., 2020). Potential explanations of subtype differences include different PD-associated protein dysfunctions (Quinn et al., 2020; Mencke et al., 2021; Menozzi and Schapira, 2021), different accumulation rates of pathogenic alpha-synuclein (Lawton et al., 2022), different trajectories of pathology progression (De Pablo-Fernández et al., 2019) or possibly even different compensatory capacities in the neural circuits (Palermo et al., 2020; Wang et al., 2022). Future studies should focus on understanding the underlying disease pathophysiology that drives these different clinical clusters in EOPD and their subsequent progression.

This study has some limitations. Our cluster analysis solution classified EOPD based on clinical presentation only, and additional variables (i.e., genetics, neuroimaging markers, and other biomarkers) may be able to further refine the clusters. Secondly, our study has the heterogeneity of clinical data collection and quality from different investigators. Nevertheless, to retain the consistency and quality of the data as much as possible, all the researchers involved in the study received standardized and unified training. Thirdly, the follow-up data are incomplete, and the reasons of causing this are varied. For example, the follow-up time of some patients with EOPD has not been reached, and the impact of COVID-19 containment measures. Moreover, the prognostic value and applicability of our recommended EOPD subtyping method should be confirmed in other cohorts.

In summary, we developed three clinical EOPD subtypes based on the cluster analysis of a large EOPD cohort. Motor symptoms, motor complications, and NMSs were distinguished among the clusters, some of which were verified in follow-up data. These findings improve our understanding of EOPD heterogeneity. Exploring EOPD subtypes will shed light on precision medicine and develop more effective approaches for clinical trials and treatment strategies for patients with EOPD in the future.

## Conclusion

We identified three different clinical subtypes (mild, intermediate, and severe) using cluster analysis in a large EOPD cohort for the first time, which is important for tailoring therapy to individuals with EOPD.

## Data availability statement

The raw data supporting the conclusions of this article will be made available by the authors, without undue reservation.

## Ethics statement

The studies involving human participants were reviewed and approved by the Ethics Committee of Xiangya Hospital of Central South University. The patients/participants provided their written informed consent to participate in this study.

## Author contributions

ZZ and XZ: research project conception, organization, execution, statistical analysis design and execution of the study, patients’ enrollment and follow up, and writing of the first manuscript draft. YX, YZ, HP, and JW: research project execution and patients’ enrollment and follow up. QX, YC, QS, XY, JG, and BT: research project organization, execution, and manuscript review and critique. XYW, JZ, XHW, and JL: statistical analysis design, execution, review and critique of the study, and manuscript review and critique. LL and ZL: research project conception, organization, execution, and manuscript review and critique. All authors contributed to the article and approved the submitted version for publication.

## Footnotes

1
http://www.pd-mdcnc.com

2
https://www.r-project.org
