# Supplementary material for: Subtyping of early-onset Parkinson’s disease using cluster analysis: A large cohort study
Source: Front Aging Neurosci. 2022 Nov 11;14:1040293. doi: 10.3389/fnagi.2022.1040293 (PMC9692000; doi:10.3389/fnagi.2022.1040293)
Supplement: Supplementary file 1 [file Data_Sheet_1.docx]

Supplementary Material

## Supplementary Figures

**
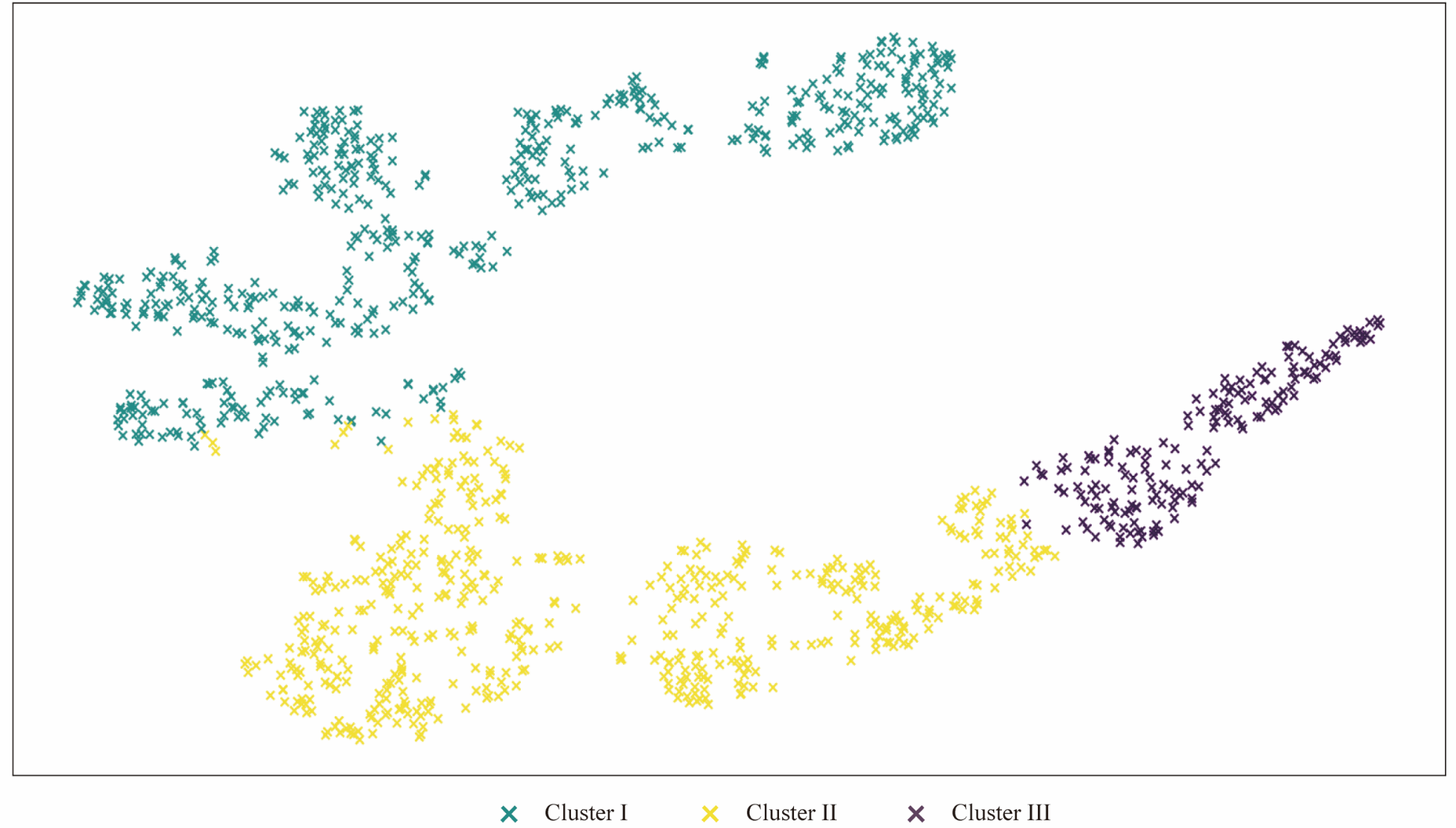
**

**Supplementary Figure 1. Visualization of the final hierarchical cluster solution in the EOPD cohort.** Cluster Ⅰ: mild motor and non-motor dysfunction, Cluster Ⅱ: intermediate, Cluster Ⅲ: severe motor and non-motor dysfunction.


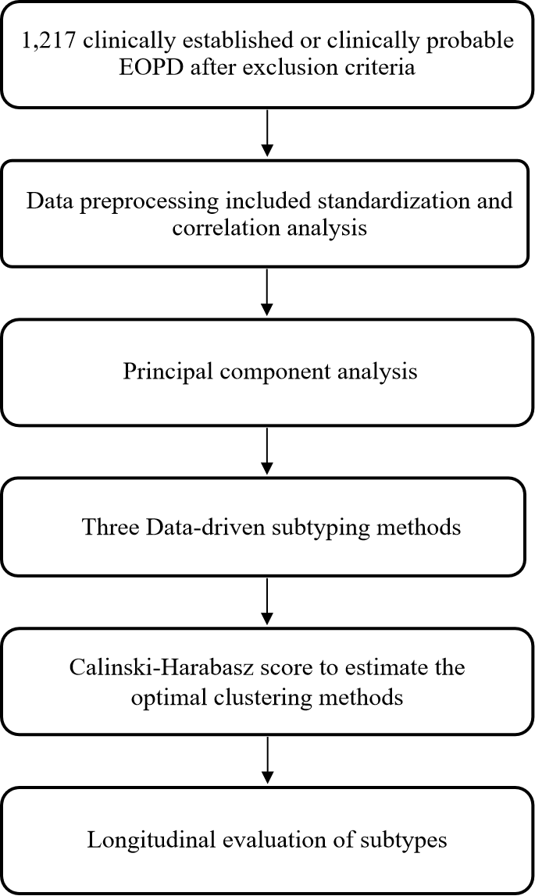


**Supplementary Figure 2. Flowchart of data-driven clustering.**

**
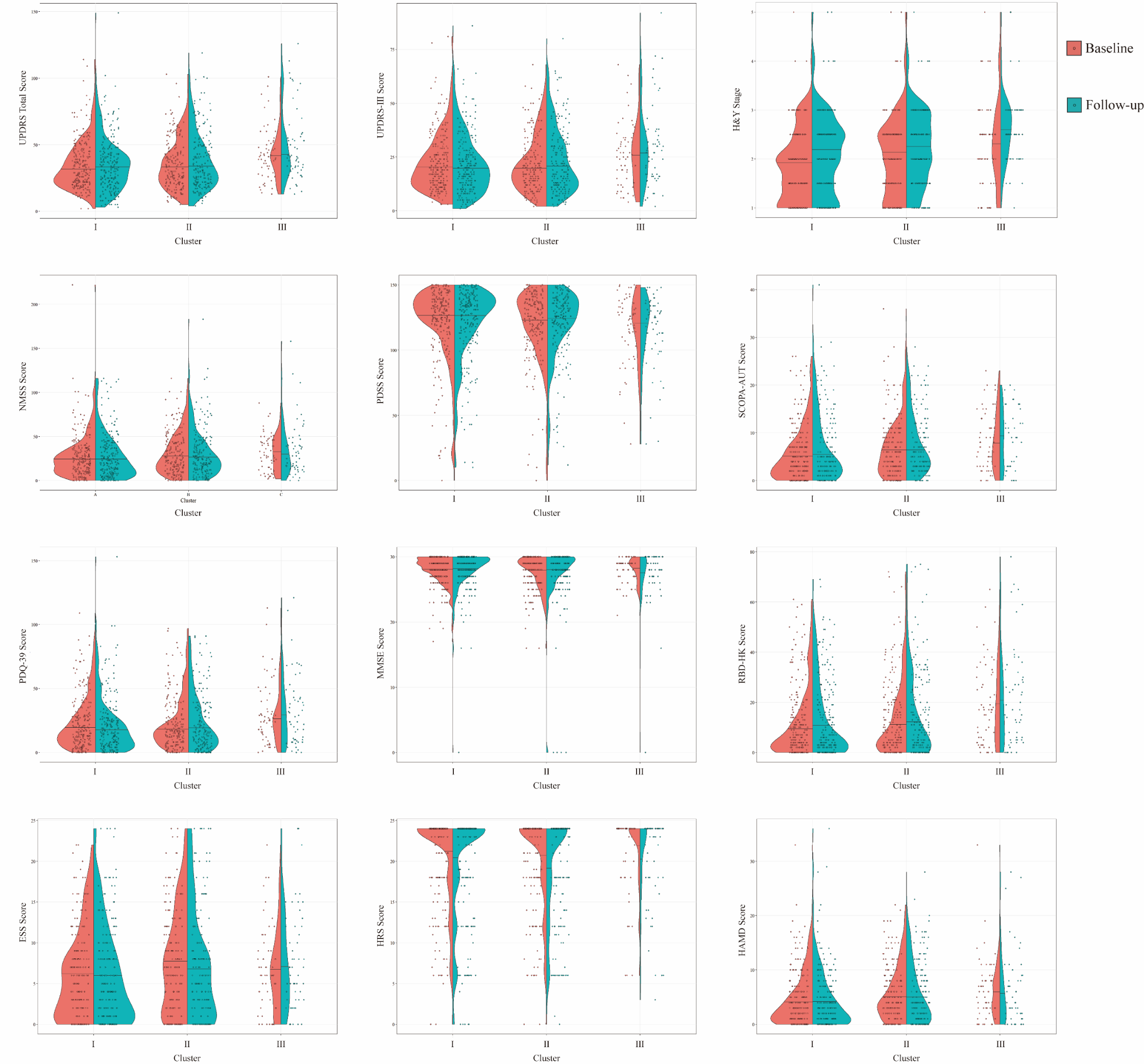
Supplementary Figure 3. Data distribution of three clusters at baseline and follow-up.**

**
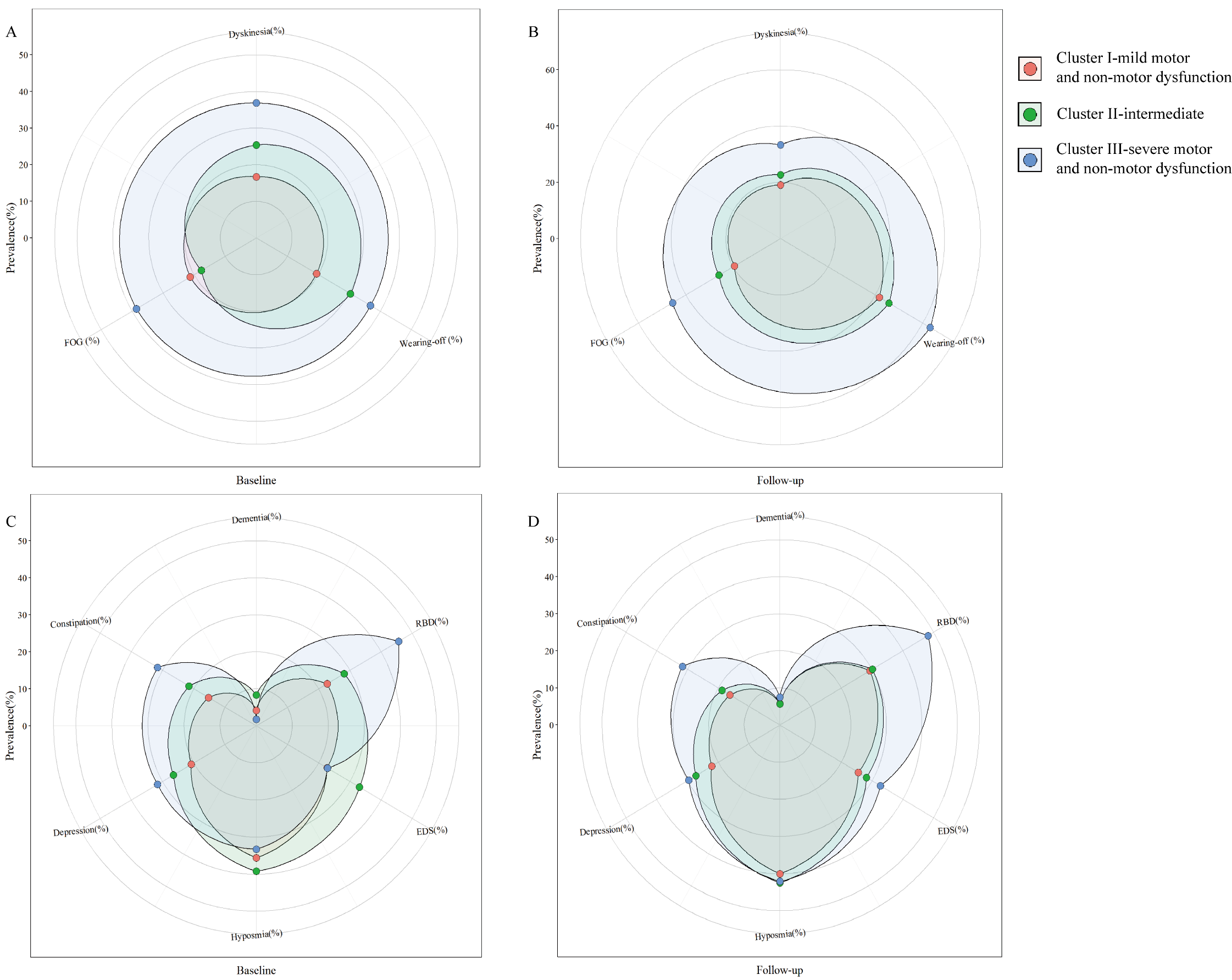
Supplementary Figure 4. Prevalence of motor and non-motor symptoms at baseline and follow-up.** (A) Prevalence of motor symptoms at baseline; (B) Prevalence of motor symptoms at follow-up; (C) Prevalence of non-motor symptoms at baseline; (D) Prevalence of non-motor symptoms at follow-up.

## Supplementary Tables

**Supplementary Table 1. Calinski-Harabasz score of three subtyping methods.**

| **Method** | **Score** |
| --- | --- |
| K-means clustering | 1939.12 |
| Hierarchical clustering | 2977.53 |
| Spectral clustering | 3047.52 |

**Supplementary Table 2. Data distribution of three subtyping methods.**

| **Method** | **Cluster Ⅰ** | **Cluster Ⅱ** | **Cluster Ⅲ** |
| --- | --- | --- | --- |
| K-means clustering | 551 | 494 | 172 |
| Hierarchical clustering | 533 | 512 | 172 |
| Spectral clustering | 668 | 533 | 16 |

**Supplementary Table 3. Baseline demography and characteristics of the EOPD cohort.**

| **Characteristics** | **Total (n=1,217)*** | **Median**  (25% ~ 75% interquartile) |
| --- | --- | --- |
| Age at baseline | 50.54±6.82 | 51.00(47.00～54.00) |
| Gender ratio (male, %) | 53.66 | - |
| BMI | 22.59±3.04 | 22.59(20.58～24.44) |
| Age at onset | 44.12±5.54 | 45.00(41.00～48.00) |
| Disease duration | 6.34±5.22 | 5.00(2.50～9.00) |
| LEDD | 317.42±238.54 | 300.00(150.00～400.00) |
| UPDRS part Ⅰ | 2.28±1.98 | 2.00(1.00～3.00) |
| UPDRS part Ⅱ | 10.98±6.56 | 10.00(7.00～14.00) |
| UPDRS part Ⅲ | 25.42±15.73 | 22.00(14.00～34.00) |
| UPDRS total score | 40.58±23.25 | 36.00(24.00～53.00) |
| Motor subtypes | 26.29/17.75/55.96 | - |
| TD/Indeterminate/PIGD (%) |  |  |
| H&Y stage (%) | 2.0 (1.5~2.5) | - |
| 1 | 19.64 | - |
| 1.5 | 16.52 | - |
| 2 | 21.28 | - |
| 2.5 | 18.41 | - |
| 3 | 18.41 | - |
| 4 | 4.11 | - |
| 5 | 1.64 | - |
| Dyskinesia (%)^a^ | 19.06 | - |
| Wearing-off (%)^b^ | 27.36 | - |
| FOG (%)^c^ | 27.12 | - |
| NMSS | 31.12±27.58 | 25.00(11.00～43.00) |
| SCOPA-AUT | 6.45±5.96 | 6.00(2.00～8.00) |
| PDSS | 119.40±26.53 | 125.00(107.00～138.00) |
| PDQ-39 | 27.84±24.87 | 21.00(9.00～39.00) |
| MMSE (mean ± SD, %)^d^ | 27.45±2.85, 8.46 | 28.00(26.00～29.00) |
| RBDQ-HK (mean ± SD, %)^e^ | 12.62±14.92, 26.46 | 6.00(1.50～19.00) |
| ESS (mean ± SD, %)^f^ | 6.55±5.83, 25.72 | 6.00(2.00～10.00) |
| HRS (mean ± SD, %)^g^ | 20.65±5.58, 33.77 | 24.00(18.00～24.00) |
| HAMD (mean ± SD, %)^h^ | 5.62±5.55, 27.44 | 4.00(1.00～8.00) |
| Constipation (%)^i^ | 18.57 | - |

*Quantitative data were expressed as mean ± SD, categorical variables were expressed as percentages, unless otherwise indicated.

a-i Evaluated respectively by UPDRS part Ⅳ-A, the 9-item End-of-dose Wearing-off Questionnaire (WOQ-9), New Freezing of Gait Questionnaire (NFOGQ), Mini-Mental State Examination (MMSE), Rapid Eye Movement Sleep Behavior Disorder Questionnaire-Hong Kong (RBDQ-HK), Epworth Sleepiness Scale (ESS), Hyposmia Rating Scale (HRS), Hamilton Depression Scale (HAMD-17) and Rome III criteria.

EOPD, Early-onset Parkinson’s Disease; BMI, Body Mass Index; LEDD, Levodopa Equivalent Daily Dose; UPDRS, Unified Parkinson’s Disease Rating Scale; TD, Tremor-Dominant; PIGD, Postural Instability and Gait Difficulty; H&Y, Hoehn and Yahr; FOG, freezing of gait; NMSS, Non-Motor Symptoms Scale; SCOPA-AUT, the Scale for Outcomes in Parkinson Disease for Autonomic Dysfunction; PDSS, Parkinson’s disease Sleep Scale; PDQ-39, Parkinson’s disease questionnaire-39 item version; MMSE, Mini Mental State Examination; RBDQ-HK, Rapid Eye Movement Sleep Behavior Disorder Questionnaire-Hong Kong; ESS, Epworth Sleepiness Scale; HRS, Hyposmia Rating Scale; HAMD, Hamilton Depression Scale.
